# Otitis media with effusion in children: Cross-frequency correlation in pure tone audiometry

**DOI:** 10.1371/journal.pone.0221405

**Published:** 2019-08-22

**Authors:** Ann Hiu Ching Chow, Ting Cai, Bradley McPherson, Feng Yang

**Affiliations:** 1 Division of Speech and Hearing Sciences, Faculty of Education, University of Hong Kong, Hong Kong, China; 2 Department of Speech Therapy, Shenzhen Children’s Hospital, Shenzhen, China; University of Palermo, ITALY

## Abstract

Different guidelines are adopted in clinics and countries to assess pure tone hearing sensitivity in children with otitis media with effusion (OME). Some guidelines specify a broad range of audiometric frequencies that must be tested and from which average thresholds determined, while others leave test frequencies unspecified. For guidelines that suggest specific frequencies there are various pure tone frequencies and frequency ranges given. The present study investigated whether (1) a full range of audiometric frequencies is required to evaluate hearing loss caused by OME in children, or if neighboring frequencies provide essentially the same threshold information, and (2) if different combinations of test frequency pure tone averaging calculations may affect decision criteria for surgical treatment. In a retrospective cohort study, right and left ear air conduction pure tone threshold data were obtained, from 125 Hz to 8 kHz, for 96 children with OME aged 4 to 12 years. Paired t-tests, correlation tests (Pearson’s r, Cronbach’s alpha, intraclass correlation) and absolute differences were used to examine the relationships among pure tone audiometric (PTA) frequencies for all ears with hearing loss. 168 ears were found to have OME-related hearing loss. Only the 125 Hz—250 Hz comparison showed no statistically significant difference between neighboring thresholds. However, only the 4 kHz and 8 kHz comparison showed a clinically significant mean difference of ≥ 10 dB. When viewing individual differences, comparison between 250 Hz and 500 Hz, 125 Hz and 500 Hz, and 4 kHz and 8 kHz, showed a large number of ears with clinically significant differences between test frequencies. Comparisons among low frequency 3 PTA average (500 Hz, 1 kHz, 2 kHz), high frequency 3 PTA average (1 kHz, 2 kHz, 4 kHz), and 4 frequency PTA average (500 Hz, 1 kHz, 2 kHz, 4 kHz) showed no statistically significant differences, with very strong correlations for all comparisons. In addition, for all the combinations of PTA averages, no clinically significant differences were found for the various comparisons or among individual results. Clinically, testing hearing sensitivity in the 125 Hz to 8 kHz range is worthwhile in evaluating hearing sensitivity in children with OME due to large individual variability across audiometric frequencies. However, frequencies tested for criterion averages for surgical treatments of children with OME may be restricted to 3 frequency PTA averages, either an average of 500 Hz, 1 kHz, 2 kHz or an average of 1 kHz, 2 kHz, 4 kHz, as no clinically significant differences were found using these or a 4 frequency averaging technique. For research purposes, 250 Hz can proxy for hearing thresholds at 125 Hz; and the low frequency 3 PTA average, high frequency 3 PTA average and 4 frequency PTA average may be used interchangeably, as no statistically significant differences were found among these measures.

## Introduction

Otitis media is a term for a spectrum of middle ear disease, with acute otitis media and otitis media with effusion being a very common illnesses among paediatric patients [1–3]. In Australia, between AUD 100 and 400 million was spent in treating otitis media in 2008 [4], and USD 497 million was spent in South Korea in 2012 [5]. The estimated total costs for otitis media in Portugal were EUR 334 million in 2009 [6]. Apart from major financial costs internationally noted in treating otitis media, considerable physical, social and emotional burdens may be experienced by the affected children and their caregivers [7].

Acute otitis media (AOM) is an infection of the middle ear caused by bacteria or viruses with a rapid onset [8,9]. Symptoms includes fever and otalgia [9]. Otitis media with effusion (OME) is defined as fluid in the middle ear with no acute inflammation symptoms [8–9]. In many cases, the only symptom of OME is fluctuating hearing status [8]. OME is often undetected until doctors raise suspicion of it or not until a child shows delayed speech development or presents with behavioural or attentional problems [8,10]. To assess hearing sensitivity in children with OME, pure tone audiometry is a key assessment tool.

The presence of fluid in the middle ear results in increasing stiffness and mass of the middle ear system, which affects the transmission of sound from the outer ear to the inner ear, resulting in conductive hearing loss [11]. Hearing levels associated with OME can vary from within normal limits to moderate hearing loss (41 dB HL– 60 dB HL) [12]. Studies have found 3 frequency air conduction pure tone audiometric (PTA) averages (500 Hz, 1 kHz, 2 kHz) in children with OME ranging from 18 dB HL to 35 dB HL [13]. A diagnostic evaluation on the hearing sensitivity of an individual using pure tone audiometry typically involves testing from 250 Hz to 8 kHz, and when a low frequency hearing loss is present, 125 Hz may also be tested [14]. Hearing thresholds at 250 Hz and 8 kHz are sometimes omitted when testing children with OME due to children’s attentional limitations [15]. Some studies and guidelines adopt the strategy of testing hearing sensitivity only in the main speech frequency range, 500 Hz to 4 kHz, when assessing children [13,16]. Testing over a limited frequency range reduces test time and hence makes attention less of an issue. However, across studies in a systematic review which investigated audiometric results in children with OME, the majority of studies found that the frequencies with the most prominent hearing loss occur at the extremes of the range of frequencies examined [13]. This raises the question of whether testing a wide range of frequencies, e.g. from 125 Hz to 8 kHz, is necessary in evaluating hearing loss in children with OME. If no significant differences exist across adjacent frequencies then hearing sensitivity in children with OME may be adequately assessed using a reduced frequency range.

Studies indicate that approximately 28% of newly diagnosed OME cases recover after 3 months without treatment and 75% of OME as a sequela of AOM also recovered spontaneously by 12 weeks [17]. According to many clinical guidelines, a 3-month active observation period is recommended before the implementation of intervention [8, 9]. During that period the child’s auditory learning environment should be optimized, for example by arranging preferential seating and rephrasing sentences to clarify ideas [9]. If OME resolved spontaneously by 3 months, non-at-risk children may be discharged whereas children at risk of OME, such as those with speech or academic delay, cleft palate, craniofacial abnormalities and Down syndrome should have their hearing monitored annually [8,9,18].

If OME persists for 3 months or more (classified as chronic OME) only around 30% of the cases may demonstrate bilateral spontaneous resolution after 12 months [17]. Therefore, depending on hearing levels, different interventions may be performed with children with chronic OME, including surveillance every 3 to 6 months if hearing levels remain normal, myringotomy with the insertion of grommets (ventilation tubes) if hearing loss persists (see Table 1) and amplification device(s) prescribed if there are contraindications for surgical treatment [8,9].

**Table 1:** Guidelines for surgical treatments in children with persistent otitis media with effusion of more than 3 months duration.

Organizations/ Countries	Average degree of hearing loss	PTA Frequencies
American Academy of Otolaryngology- Head and Neck Surgery Foundation (AAO-HNSF) ^a^ [9]	Did not specify ^b^	Did not specify
British Columbia Medical Association [19]	Did not specify	Did not specify
Danish Health and Medicines Authority (DHMA) & Danish Society of Otorhinolaryngology, Head and Neck Surgery [20]	25 dB HL or worse	Did not specify
Darwin Otitis Guidelines Group and the Office for Aboriginal and Torres Strait Islander Health Otitis Media Technical Advisory Group [21]	Worse than 20 dB HL in both ears	500 Hz, 1 kHz, 2 kHz
Development Group of Management of Otitis Media with Effusion, Malaysia [22]	Worse than 25 dB HL ^c^	3 frequencies (frequencies were not specified)
Herefordshire Clinical Commissioning Group [23]	25 dB HL or worse in the better ear ^d^	500 Hz, 1 kHz, 2 kHz, 4 kHz
Japan Otological Society & Japan Society for Pediatric Otorhinolaryngology [24]	40 dB HL or worse in both ears ^e^	Did not specify
Korean Society of Otology [18]	40 dB HL or worse in both ears ^f^	Did not specify
National Institute for Health and Clinical Excellence (NICE) [8]	25 dB HL or worse in the better ear ^g^	500 Hz, 1 kHz, 2 kHz, 4 kHz
North West London Collaboration of Clinical Commissioning Groups [25]	25 dB HL or worse ^h^	Did not specify
Scottish Intercollegiate Guidelines Network (SIGN) [26]	Did not specify	Did not specify

^a^ Co-developed with the American Academy of Pediatrics and the American Academy of Family Physicians.

^b^ Surgery may be offered if the child has bilateral hearing loss worse than 20 dB HL at 1 or more frequencies (500 Hz, 1 kHz, 2 kHz, 4 kHz).

^c^ Surgery may be considered if there are middle ear pathologies.

^d^ Surgery may also be considered if the child has at least 5 recurrences of acute otitis media in one year or has bilateral persistent OME with hearing loss less than 30 dB HL that is impacting the child’ development.

^e^ Surgery may be recommended if the child has an average of 25–39 dB HL bilateral hearing loss or if eardrum pathology is observed irrespective of degree of hearing loss.

^f^ Surgery may be recommended if the child has an average of 20–39 dB HL bilateral hearing loss or unilateral OME.

^g^ Surgery may be considered if the child has developmental, social or educational problems irrespective of degree of hearing loss.

^h^ Surgery may also be considered if the child has at least 5 recurrences of acute otitis media in one year or presents with speech delay or behavioural problems related to associated hearing impairment or with other health problems, such as Down syndrome or cleft palate.

As shown in Table 1, the PTA frequency criteria for surgical treatments in children with chronic OME differ among expert groups. For example, some use a 3 PTA frequency average to calculate overall PTA average due to the importance of those frequencies in speech recognition, some also include 4 kHz in the average to acknowledge the importance of higher frequencies for speech intelligibility [27]. The inconsistency in the frequencies chosen raises concern if the selection of the PTA frequencies can affect the average hearing level calculation outcome and therefore the criteria used for surgical intervention. Whether the differences among the averages are significantly different from a clinical perspective also requires evaluation. To the authors’ knowledge, no studies have explored this issue in a systematic manner.

In summary, the aims of the present study were: (1) to investigate the correlation between 125 Hz and 250 Hz, 250 Hz and 500 Hz, 125 Hz and 500 Hz (a near-neighboring low frequency pair), and between 4 kHz and 8 kHz when assessing hearing levels in children with otitis media with effusion, to understand how much of the variation in one frequency is related to an adjacent frequency; (2) to evaluate the degree of relationship among hearing threshold average calculations, namely in the overall pure tone average (125 Hz– 8kHz), low frequency 3 PTA average (500 Hz, 1 kHz, 2 kHz), high frequency 3 PTA average (1 kHz, 2 kHz, 4 kHz) and 4 frequency PTA average (500 Hz, 1 kHz, 2 kHz, 4 kHz) in children with OME-related hearing loss, to determine the degree of relationship among PTA averages; and (3) to determine whether any differences among relationships noted in (1) and (2) were clinically significant, with a clinically significant difference defined as a difference between compared thresholds of ≥10 dB.

## Materials and methods

### Subjects

Children aged from 4 to 12 years, who were diagnosed with otitis media with effusion, were assessed during December 2014 to August 2015. All participants were recruited from the Department of Otorhinolaryngology—Head and Neck Surgery, Shenzhen Children’s Hospital, China. Children attended the clinic due to parental concern regarding hearing loss or for follow-up appointments after acute otitis media or OME. Parents provided informed written consent approving their child’s participation in the research program. Children with unilateral/bilateral mixed hearing loss, with ventilation tubes in situ, and those who were unable to provide a full range of pure tone audiometry frequency thresholds were excluded from the present study. A sample size of n ≥ 133 ears was targeted, to achieve a power of 80% and a significance level of 1% for detecting an effect size of 0.3 between pairs. The present study extends previously published work on audiometric profiles with the same cohort [28] and used retrospective anonymized clinical data analyzed during January to April 2018. The Faculty Research Ethics Committee, Faculty of Education, University of Hong Kong approved the study protocol (January 12, 2018).

### Equipment

A portable otoscope (Welch-Allyn Inc., Skaneateles Falls, NY) was used to examine for clinical signs of OME. A 226 Hz tympanometer (TympStar, GSI, Eden Prairie, MN) at 85 dB SPL with sweep rate of 50 daPa/s, calibrated to ANSI S3.39–1987 (R 2007) standards [29] was used to aid identification of each child’s middle ear status. A pure tone audiometer (204A, Entomed, Svedala, Sweden) using insert (ER-3A, Etymotic, Elk Grove Village, IL) earphones in a sound booth, with ambient noise within acceptable limits [30] was used to test the hearing thresholds in each child. The audiometer/earphones were calibrated to ISO standards [31].

### Procedure

All participants were identified with otitis media with effusion through otoscopic examination, tympanometry and ipsilateral acoustic reflex assessment by an ENT specialist. Children with diagnosed otitis media with effusion were further tested for their hearing sensitivity with pure tone audiometry. Clinical otoscopic signs of OME included retracted tympanic membrane, foreshortened handle of malleus, absence of cone of light, discoloration of tympanic membrane, and observable bubbles and fluid levels [32]. Jerger’s classifications of type B, indicating a flat tympanogram without prominent peak, and type C2, with negative middle ear pressure between -200 daPa to -350 daPa, were considered to be consistent with middle ear effusion [33]. An absence of 1000 Hz ipsilateral acoustic reflexes at 105 dB SPL maximum stimulus level, in addition to indicative results from otoscopic examination and tympanometry, led to an OME diagnosis.

### Pure tone audiometry

Children with diagnosed OME had their hearing sensitivity tested with pure tone audiometry. Air conduction pure tone thresholds at 125 Hz, 250 Hz, 500 Hz, 1 kHz, 2 kHz, 4 kHz, and 8 kHz were tested. Bone conduction thresholds (250 Hz to 4 kHz) were obtained only if the air conduction hearing thresholds were worse than 20 dB HL. Children with bone conduction thresholds of ≥ 25 dB HL at 500 Hz, 1 kHz or 4 kHz were considered as having a sensorineural hearing loss component to the hearing loss and excluded from the study. 2 kHz bone conduction thresholds were not included as a consistent 2 kHz bone conduction dip is often shown in the pure tone audiogram of children with otitis media with effusion [28].

### Data analysis

Pure tone frequency comparisons and pure tone average comparisons were analyzed at both group and individual levels. The pure tone frequency comparisons that were investigated were 125 Hz and 250 Hz; 250 Hz and 500 Hz, 125 Hz and 500 Hz, and 4 kHz and 8 kHz. The pure tone average comparisons examined were the overall PTA average (125 Hz– 8 kHz) with the low frequency 3 PTA average (500 Hz, 1 kHz, 2 kHz); the overall PTA average with the high frequency 3 PTA average (1 kHz, 2 kHz, 4 kHz); the overall PTA average with the 4 frequency PTA average (500 Hz, 1 kHz, 2 kHz, 4 kHz); the 4 frequency PTA average with the low frequency 3 PTA average; the 4 frequency PTA average with the high frequency 3 PTA average; and the low frequency 3 PTA average with the high frequency 3 PTA average. Group level analysis took the average of all the OME ears in the data set and individual level analysis examined the various comparisons in each participant ear. The comparisons were selected (a) in view of their saliency to typical OME audiometric configurations [13], hence the high frequency average included 1000 Hz, where thresholds are often adversely affected, and (b) without reference to inter-octave frequencies, which are often not assessed in young children.

For group level comparisons, paired t-tests were performed to investigate statistically significant differences. An alpha level of p < 0.01 was used to determine statistical significance. Clinically significant differences were also determined for the mean differences. In usual clinical practice, threshold measurements are determined in 5 dB steps [34]. When the test-retest hearing threshold does not differ by more than 5 dB, the hearing threshold at that frequency is typically regarded as a reliable response [14]. Some studies defined less than 10 dB as no significant threshold change [35]. Others defined 5 dB intra-frequency change as a measurement error that is not clinically significant [34]. Therefore, the present study examined the absolute individual hearing threshold differences at different frequency comparisons using an analogous clinically significant difference criterion of ≥ 10 dB. When more than 10% of OME ears were outside of the limit of agreement (LOA) range, it is considered as a clinically significant number of ears beyond the selected criterion.

More than one estimation of agreement is recommended as no one measure is definitive [36]. Therefore, in the present study, three different correlation tests were adopted. Pearson’s r, Cronbach’s alpha and the intraclass correlation were derived at the group level to determine the degree of relationship between test frequencies of interest. Pearson’s r gives the degree of relationship between two groups [37]. Cronbach’s alpha assesses the reliability of scale measures [38]. Intraclass correlation gauges the consistency of separate measures of the same essential entity [37]. The correlation coefficient values considered to show a strong correlation between groups were ≥ 0.68, ≥ 0.7 and ≥ 0.6 for Pearson’s r, Cronbach’s alpha and intraclass correlation, respectively [39–41]. Cohen’s d was used to determine the effect size for statistically significant findings. Effect size were categorized into small (d = 0.2), medium (d = 0.5) and large (d = 0.8) effects [42].

At the individual analysis level, Bland-Altman analysis was performed to indicate absolute threshold differences among individuals in each comparison [43]. The percentage of OME ears outside of the limit of agreement (LOA) range for a clinically significant difference of ≥ 10 dB were calculated. The percentage of OME ears outside the 95% LOA range were also determined. If more than 10% of the OME ears were beyond the limit of agreement range, this was regarded as a significant number of ears showing substantial individual differences.

## Results

Ninety-six children (age range = 4–12 years; mean = 7.83 years; 45 females) were diagnosed with otitis media with effusion (OME). Seventy-two children had bilateral OME and 24 had unilateral OME. A total of 168 ears were diagnosed with otitis media with effusion.

A paired t-test for bilateral OME ears and an independent t-test for unilateral OME ears was performed to check for statistically and clinically significant differences between right and left ears in each frequency with a 99% confidence interval. A rigorous confidence interval was chosen in view of the large sample sizes and as only clinically minor differences between ears were observed in the data set. Only 250 Hz in the bilateral OME condition showed a statistically significant difference between right and left ears (t = 3.56, df = 71, p = 0.001) and no frequencies showed clinically significant differences between the ears, i.e., all had < 10 dB HL differences. No frequencies in the unilateral OME comparison showed clinically or statistically significant differences. Therefore, right and left ears were combined for the following analyses. The Shapiro-Wilk test in addition to means, standard deviations, skewness and kurtosis values were used to check for the normality of all threshold data sets. All threshold frequencies and PTA averages were considered to be normally distributed and parametric tests were therefore used in all analyses.

### Pure tone frequency comparisons

#### Statistical and clinical significance

Averaging across all ears, thresholds of 28 dB HL at 125 Hz, 29 dB HL at 250 Hz, 27 dB HL at 500 Hz, 27 dB HL at 4 kHz and 39 dB HL at 8 kHz were found. Paired t-tests were performed between 125 Hz and 250 Hz; 250 Hz and 500 Hz; 125 Hz and 500 Hz; and 4 kHz and 8 kHz. The mean differences between the frequency comparisons are shown in Table 2. Only the comparison between 125 Hz and 250 Hz was not statistically significant and only the 4 kHz and 8 kHz comparison showed a clinically significant difference.

**Table 2:** Average mean differences, t-values, degrees of freedom, p-values and Cohen’s d for paired t-test of the frequencies of interest.

Frequencies	Mean difference (dB HL)	t-value	degrees of freedom	p-value	Cohen’s d
125 Hz and 250 Hz	-0.5	1.352	167	0.178	0.1
250 Hz and 500 Hz	1.9	4.055	167	0.000	0.3
125 Hz and 500 Hz	1.4	2.645	167	0.009	0.2
4 kHz and 8 kHz	- 11.3	- 13.780	167	0.000	1.1

#### Correlation tests

Correlation tests consistently showed a strong positive correlation in all conditions. The coefficients of Pearson’s r, Cronbach’s alpha and intraclass correlation for the different frequency conditions are shown in Table 3.

**Table 3:** Correlation coefficients—Pearson’s r, Cronbach’s alpha and intraclass correlation—for the frequencies of interest.

Frequencies	Pearson’s r	Cronbach’s alpha	intraclass correlation
125 Hz and 250 Hz	0.866	0.928	0.927
250 Hz and 500 Hz	0.799	0.888	0.879
125 Hz and 500 Hz	0.739	0.849	0.845
4 kHz and 8 kHz	0.718	0.835	0.704

#### Individual frequency differences

Absolute differences for the thresholds of the frequencies of interest among all the ears with OME were calculated with Bland-Altman analyses. The percentage of ears outside of the limit of agreement (LOA) range for clinically significant differences of ≥ 10 dB and the percentage of ears outside of the statistical 95% LOA range for the frequencies of interest are shown in Table 4.

**Table 4:** Percentage of ears outside of the limit of agreement (LOA) range for clinically significant differences of ≥ 10 dB HL and the percentage of ears outside of the statistical 95% LOA range for the frequencies of interest.

Frequencies	Percentage of ears outside of the LOA range for a clinically significant difference of ≥ 10 dB HL	Percentage of ears outside of the statistical 95% LOA range
125 Hz and 250 Hz	7%	4%
250 Hz and 500 Hz	20%	5%
125 Hz and 500 Hz	28%	7%
4 kHz and 8 kHz	63%	4%

### Pure tone average comparisons

#### Statistical and clinical significance

The various PTA averages for all ears were determined. These were found to be 29 dB HL in the overall PTA average, 27 dB HL in the low frequency 3 PTA average, 27 dB HL in the high frequency 3 PTA average, and 27 dB HL in the 4 frequency PTA average. Mean differences between averages and paired t-tests results for all PTA averaging combinations, including overall PTA average (125 Hz– 8 kHz), low frequency 3 PTA average (500 Hz, 1 kHz, 2 kHz), high frequency 3 PTA average (1 kHz, 2 kHz, 4 kHz), and 4 frequency PTA average (500 Hz, 1 kHz, 2 kHz, 4 kHz) are shown in Table 5. Three of the comparisons—overall PTA versus low frequency 3 PTA, high frequency PTA, and 4 frequency PTA averages—were found to show significant differences.

**Table 5:** Average mean differences, t-values, degrees of freedom and p-values for paired t-tests of different PTA average combinations.

PTA averages	Mean difference (dB HL)	t-value	degrees of freedom	p-value	Cohen’s d
Overall PTA average and Low frequency 3 PTA average	2.4	9.874	167	0.000	0.8
Overall PTA average and High frequency 3 PTA average	2.2	7.963	167	0.000	0.6
Overall PTA average and 4 frequency PTA average	2.1	10.203	167	0.000	0.8
4 frequency PTA average and Low frequency 3 PTA average	0.3	1.916	167	0.057	0.1
4 frequency PTA average and High frequency 3 PTA average	0.1	1.072	167	0.285	0.0
Low frequency 3 PTA average and High frequency 3PTA average	-0.2	- 0.661	167	0.510	0.0

#### Correlation tests

Correlations among the combinations of PTA averages were analyzed using Pearson’s r, Cronbach’s alpha and intraclass correlation procedures. All correlation coefficients were > 0.9, indicating very strong positive correlations for all examined combinations of averages (Table 6).

**Table 6:** Correlation coefficients of Pearson’s r, Cronbach’s alpha and intraclass correlation with different PTA average combinations.

PTA averages	Pearson’s r	Cronbach’s alpha	intraclass correlation
Overall PTA average and Low frequency 3 PTA average	0.948	0.973	0.958
Overall PTA average and High frequency 3 PTA average	0.947	0.968	0.957
Overall PTA average and 4 frequency PTA average	0.967	0.982	0.972
4 frequency PTA average and Low frequency 3 PTA average	0.978	0.988	0.988
4 frequency PTA average and High frequency 3 PTA average	0.989	0.993	0.993
Low frequency 3 PTA average and High frequency 3 PTA average	0.952	0.972	0.972

#### Individual differences

Absolute differences in each ear were derived using Bland-Altman analyses. The percentage of ears outside of the LOA range for clinically significant differences of ≥ 10 dB and the percentage of ears outside of the statistical 95% LOA range of the PTA average comparisons are shown in Table 7.

**Table 7:** Percentage of ears outside the limit of agreement (LOA) range for a clinically significant difference of ≥ 10 dB and percentage of ears outside of the statistical 95% LOA range for PTA average comparisons.

PTA averages	Percentage of ears outside of the LOA range for a clinically significant differences of ≥ 10 dB HL	Percentage of ears outside of the statistical 95% LOA range
Overall PTA average and Low frequency 3 PTA average	2%	4%
Overall PTA average and High frequency 3 PTA average	1%	7%
Overall PTA average and 4 frequency PTA average	0%	7%
4 frequency PTA average and Low frequency 3 PTA average	0%	5%
4 frequency PTA average and High frequency 3 PTA average	0%	5%
Low frequency 3 PTA average and High frequency 3 PTA average	2%	7%

## Discussion

### Pure tone frequency comparisons

Frequency correlations between 125 Hz and 250 Hz, 250 Hz and 500 Hz, 125 Hz and 500 Hz, and 4 kHz and 8 kHz, were investigated to examine whether a full range of frequencies, 125 Hz to 8 kHz, is necessary to evaluate hearing loss in children with otitis media with effusion (OME). As shown in Table 2, averaging across all participants, results showed that the relationship between 125 Hz and 250 Hz was not statistically or clinically significant (p = 0.178; mean difference = -0.5 dB HL). Results from the comparison between 250 Hz and 500 Hz, and 125 Hz and 500 Hz, showed statistically significant differences with relatively small effect sizes and no clinically significant differences were found. Interestingly, averaging across all OME ears, for the comparison between 4 kHz and 8 kHz statistically significant differences were shown and the effect size was large, reflected in a more than 10 dB HL clinically significant mean difference between the two thresholds. Correlation tests showed high correlation for all threshold comparisons.

For the comparison between 125 Hz and 250 Hz, less than 10% of the OME ears showed absolute differences beyond the LOA range with the clinically significant criterion of ≥ 10 dB. Twenty percent and 28% of the OME ears were outside of the LOA range for clinically significant differences of ≥ 10 dB for the comparison between 250 Hz and 500 Hz, and 125 Hz and 500 Hz respectively. This suggests hearing sensitivity at 125 Hz, 250 Hz, and 500 Hz in children with OME differs considerably within individuals and that, clinically, any hearing threshold at 125 Hz, 250 Hz and 500 Hz cannot be replaced by another of the three thresholds. When comparing 4 kHz and 8 kHz at a clinical significant criterion of ≥ 10 dB, 63%—more than half the OME ears—were outside of the LOA range. This indicated that many OME ears showed large differences across neighboring frequencies. Despite high statistical correlation between 4 kHz and 8 kHz, a clinically significant difference was shown in the group level analysis, and intrasubject differences were up to 50 dB between 4 kHz and 8 kHz. Overall, this analysis suggests that at both the group and individual levels, 8 kHz cannot be replaced by 4 kHz when testing hearing sensitivity in individual children with OME.

Results revealed that hearing sensitivity at 4 kHz and 8 kHz showed the largest difference when compared with other frequency comparisons. Due to dysfunction of the Eustachian tube in OME children, stiffness in the middle ear increases. As stiffness affects the transmission of low frequency sound the most, OME at an early stage shows low frequency hearing loss. However, in later stages of OME, fluid accumulates in the middle ear, increasing the mass in the middle ear. Since high frequency sound transmission is mostly affected by mass, high frequency hearing loss then occurs [44].

A full range of PTA frequencies (125 Hz to 8 kHz), as shown from the present study, is necessary to precisely evaluate hearing sensitivity in children with OME in clinical settings. On the other hand, for research purposes, as the comparison between 125 Hz and 250 Hz showed no statistically significant differences, high correlations between the thresholds were found, and less than 10% of the OME ears were outside the statistical 95% LOA range for the 125 Hz and 250 Hz comparison, researchers can assume that 125 Hz thresholds would be statistically equivalent to 250 Hz thresholds at the group level.

### Pure tone average comparisons

The comparison between the overall PTA average with the other PTA averages (low frequency 3 PTA average, high frequency 3 PTA average, 4 frequency PTA average) found statistically significant differences. However, comparison between low frequency 3 PTA average, high frequency 3 PTA average and 4 frequency PTA average did not show statistically significant differences. Furthermore, for all PTA average combinations no clinically significant differences were found in the various comparisons in the group level analysis.

Under the group level, by averaging across all OME ears there were statistically significant differences in the comparisons of the different PTA averages. However, effect sizes for the differences were relatively small and no clinically significant differences were shown. The comparison between the 4 frequency PTA average and the 3 frequency PTA averages (low frequency 3 PTA average and high frequency 3 PTA average), and between the low frequency 3 PTA average and the high frequency 3 PTA average showed no statistically or clinically significant differences. All combinations of PTA averages showed very strong positive correlations. At the individual level of analysis, less than 10% of the OME ears in all PTA average comparisons were outside of the LOA range of a clinically significant difference of ≥ 10 dB. This indicated that only a very small number of OME ears showed substantial individual differences for different PTA average comparisons.

The above results indicate that using different combinations of test frequencies in calculating PTA average, as used in different clinical guidelines, is not likely to affect the criteria for medical/surgical treatment of children with OME. However, when evaluating the need for surgery in children with OME, the frequencies tested may be restricted to 3 frequency PTA averages, either an average of 500 Hz, 1 kHz, 2 kHz or an average of 1 kHz, 2 kHz, 4 kHz, if a reduced audiological assessment time is desired.

In addition, there were high correlations and no statistically significant differences between the 4 frequency PTA average (500 Hz, 1 kHz, 2 kHz, 4 kHz), low frequency 3 PTA average (500 Hz, 1 kHz, 2 kHz) and high frequency 3 PTA average (1 kHz, 2 kHz, 4 kHz) comparisons, and less than 10% of the OME ears were outside the 95% statistical LOA range for those PTA average comparisons in an individual level analysis. Hence, for both research and clinical purposes these averages can be validly equated. It should be noted that there are no other known studies to compare the current results to and hence replication studies that focus on similar audiometric data would be of value.

### Limitations

In the present study, all participants were recruited from the same hospital in Shenzhen and the children were all Chinese, and aged 4 to 12 years old. The peak incidence periods for OME may occur at 2 and 5 years of age [10]. Thus, the age criteria of this study may not fully reflect the hearing levels of children with OME at the age of peak prevalence, and the results from this study may not be generalizable to the whole paediatric population in China or other countries. Also, inter-octave frequencies were not tested in this present study. While the American Speech-Language-Hearing Association recommended hearing levels at 250 Hz, 500 Hz, 1 kHz, 2 kHz, 3 kHz, 4 kHz, 6 kHz and 8 kHz should be tested for diagnostic evaluation [14], the British Society of Audiology [45] suggested assessment at 3 kHz and 6 kHz should be considered if high frequency hearing loss is present. Therefore, as 3 kHz and 6 kHz were not measured in this current study and not all combinations of frequency correlations were determined, the study was not able to demonstrate frequency correlations in the entire potentially assessed frequency range for children with OME.

## Conclusions

The present study, to the best of our knowledge, is the first to investigate the relationships between PTA thresholds, and the relationships between different combinations of PTA frequencies used to guide surgical intervention and clinical decision-making in children with OME.

Results show that clinically, individuals may exhibit large variability in their hearing thresholds, especially in the high frequencies (e.g., the comparison between 4 kHz and 8 kHz), with clinically significant differences found. Therefore, testing hearing sensitivity from 125 Hz to 8 kHz is necessary to fully evaluate hearing sensitivity in individual children with OME in clinical settings. For research purposes, 250 Hz can adequately represent hearing thresholds at 125 Hz.

When evaluating the need for surgery on children with OME, hearing thresholds to be tested can be minimized to any standard 3 frequency PTA average, either an average of 500 Hz, 1 kHz, 2 kHz or an average of 1 kHz, 2 kHz, 4 kHz. In addition, as the low frequency 3 PTA average (500 Hz, 1 kHz, 2 kHz), high frequency 3 PTA average (1 kHz, 2 kHz, 4 kHz) and 4 frequency PTA average (500 Hz, 1 kHz, 2 kHz, 4 kHz) did not show statistical significant differences and less than 10% of OME ears were outside of the statistical 95% limit of agreement range, for research purposes these PTA average comparisons may be used interchangeably.

## Supporting information

S1 File Completed STROBE checklist. STROBE_checklist_v4_combined_PlosMedicine Chow et al. MS.doc.
(DOC)
